# North Sea ecosystem change from swimming crabs to seagulls

**DOI:** 10.1098/rsbl.2012.0474

**Published:** 2012-07-04

**Authors:** C. Luczak, G. Beaugrand, J. A. Lindley, J-M. Dewarumez, P. J. Dubois, R. R. Kirby

**Affiliations:** 1 Centre National de la Recherche Scientifique, LOG UMR 8187, Université Lille 1, France; 2 Sir Alister Hardy Foundation for Ocean Science, Plymouth, UK; 3 Marine Institute, Plymouth University, Plymouth PL4 8AA, UK

**Keywords:** climate change, food web, *Larus fuscus graelsii*, plankton, *Polybius henslowii*, sea temperature

## Abstract

A recent increase in sea temperature has established a new ecosystem dynamic regime in the North Sea. Climate-induced changes in decapods have played an important role. Here, we reveal a coincident increase in the abundance of swimming crabs and lesser black-backed gull colonies in the North Sea, both in time and in space. Swimming crabs are an important food source for lesser black-backed gulls during the breeding season. Inhabiting the land, but feeding mainly at sea, lesser black-backed gulls provide a link between marine and terrestrial ecosystems, since the bottom-up influence of allochthonous nutrient input from seabirds to coastal soils can structure the terrestrial food web. We, therefore, suggest that climate-driven changes in trophic interactions in the marine food web may also have ensuing ramifications for the coastal ecology of the North Sea.

## 1. Introduction

Temperature is an important driver of the trophodynamics of the North Sea ecosystem and a recent shift in temperature, in combination with overfishing, has established a new ecosystem dynamic regime through a series of internal mechanisms [1]. Many of the changes in the biology of the North Sea have been witnessed first in the plankton [2]. For example, the abundance of decapod larvae in the plankton is correlated positively with sea temperature and, as the North Sea has warmed their numbers have increased. Among the decapod larvae, swimming crabs of the subfamily Polybiinae have increased in abundance in particular, and among them the warm-water swimming crab *Polybius henslowii*, the most pelagic of the Polybiinae, has colonized the North Sea [3,4].

Adult swimming crabs are an important food for lesser black-backed gulls *Larus fuscus graelsii* during the breeding period [5,6]. Here, we examine whether climate-induced changes in swimming crabs might have influenced the abundance of lesser black-backed gulls in the North Sea, constituting a link between marine and terrestrial ecosystems. First, we study whether long-term changes in sea surface temperature (SST) and abundances in larval and adult swimming crabs are related to changes in the breeding colonies of *L. fuscus graelsii* in Northern France and Belgium. Second, we examine how changes in adult swimming crabs between 2000 and 1986 are correlated with the population growth rate of the 21 major North Sea colonies of lesser black-backed gulls.

## 2. Material and methods

### (a) North Sea surface temperature

SST data for the period 1978–2009 in the area 51° N–60° N, 4° W–10° E were obtained from the ERSST_V3 dataset (2° latitude×2° longitude) [7] and transformed as monthly anomalies by subtracting the long-term average of the corresponding month mean.

### (b) Adult swimming crabs

Data on adult swimming crabs were collected by the North Sea benthos survey in 1986 and 2000 [8], and on four to six occasions a year at Gravelines (Northern France), southern North Sea (51°01′ 40 N, 2°04′35 E) [5] from 1978 to 2009. Using the North Sea benthos survey data, we estimated changes between 1986 and 2000 by spatially interpolating the data for each year using the inverse squared distance method with a search radius of 100 km [9] on a grid 0.25° longitude×0.25° latitude (51° N–60° N, 4° W–10° E). We calculated separately the monthly abundance anomalies for the Gravelines dataset, also by using the inverse squared method [9].

### (c) Lesser black-backed gulls

Data on lesser black-backed gulls (number of breeding pairs) in Northern France (Calais, Gravelines and Dunkerque) and in Belgium (Zeebrugge and Het Zwin) for the period 1978–2009 were obtained from personal monitoring (P.J.D. and C.L.) and the literature [10,11] (see electronic supplementary material). Locations of the major North Sea breeding colonies and their annual growth rates approximately between 1986 and 2000 were also retrieved from the literature [10–14].

### (d) Decapod larvae

Decapod larvae were collected by the Continuous Plankton Recorder (CPR) survey [3] and Polybiinae larvae sampled in 2010 were identified molecularly [4]. Monthly anomalies of all decapod larvae were calculated between 1978 and 2009 for the region 51° N–60° N, 4° W–10° E.

### (e) Methods

Long-term monthly changes in decapod larvae and adult swimming crabs were examined in relation to both the annual number of *L. fuscus graelsii* breeding pairs in colonies in Northern France (Calais, Dunkerque) and in Belgium (Zeebrugge, Ostend), and annual SST anomalies between 1978 and 2009. We performed cross-correlation analyses between the annual average of SST, decapod larvae, adult swimming crabs and the number of pairs of lesser black-backed gulls in French and Belgian colonies lagging between 0 and 4 years either annual SST, or the lowest trophic level parameter among the pair of correlated variables. Probabilities were corrected to account for temporal autocorrelation [4]. All biological variables were transformed using the function log_10_(*x* + 1) to stabilize the variance in the data. Spatial changes in adult swimming crabs were investigated by subtracting the logarithm of the abundance in 2000 by 1986. We performed a paired *t*-test on benthic stations sampled in both 1986 and 2000. We estimated the magnitude of changes in swimming crabs corresponding to the maximum foraging distance of each major *L. fuscus graelsii* colony around the North Sea (135 km [15]); while this distance is mainly based upon fish discards so that the gull may have a much more restricted range of less than 40 km [16], we kept the larger radius as the number of samples was insufficient to provide an adequate estimate of values on swimming crabs. We next obtained the annual population growth rate (percentage change) of *L. fuscus graelsii* colonies around the North Sea coasts between 2000 and 1986 at both county and regional scales by applying a standard procedure [12,17]; we excluded colonies of less than10 breeding pairs. Finally, we examined the Spearman correlation between the magnitude of changes in swimming crabs at the vicinity of each major colony and the annual population growth rates of seabird colonies, testing the correlation by 1000 permutations.

## 3. Results

**Figure 1.**
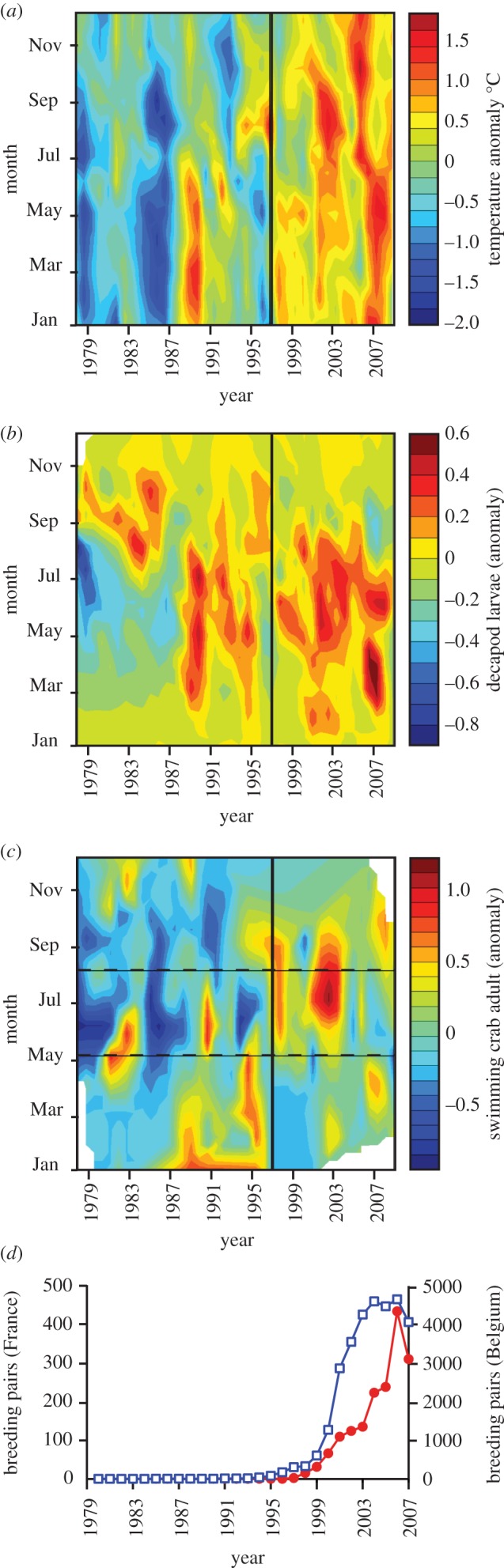
Long-term changes in SST, decapods and in lesser black-backed gulls. The vertical line separates periods before and after 1997. (*a*) Monthly SST anomalies for the period 1978–2009. (*b*) Monthly anomalies of all decapod larvae in CPR samples in the region 51° N–60° N, 4° W–10° E. (*c*) Monthly anomalies of adult swimming crabs at Gravelines. The two horizontal dashed lines indicate the breeding period of lesser black-backed gulls (*d*) Breeding pairs of lesser black-backed gulls in Northern France (filled red circles) (Calais, Gravelines and Dunkerque) and in Belgium (open blue squares) (mainly Zeebrugge, but also Het Zwin) (see figure 2*b*).

Figure 1*a* reveals two increases in North Sea SST, the first around 1989 followed by a second, sustained warming after  approximately 1997 leading to warmer SST throughout the year. Figure 1*b,c* reveal a coincident increase in decapod larvae in the North Sea and in adult swimming crabs in the benthos at Gravelines, respectively. Figure 1*d* indicates the establishment of a new colony and a substantial increase in breeding pairs of lesser black-backed gulls after 1997 in Northern France and Belgium, respectively. We found significant positive correlations between SST and decapod larvae, between decapod larvae and adult swimming crabs and between adult swimming crabs and the population growth of lesser black-backed gull colonies in both France and Belgium with either 0, a 1 or a 3–4 year lags in the relationships, respectively (table 1).

**Table 1.** Cross-correlation analyses between the annual average of SST, decapod larvae, adult swimming crabs and the number of pairs of lesser black-backed gulls in French and Belgian colonies with a lag between 0 and 4 years. Probabilities were corrected to account for temporal autocorrelation. *p < 0.05; **p < 0.01; ***p < 0.001; values in bold indicate the strongest correlation.

lag in years	SST and decapod larvae	decapod larvae and adult swimming crabs	adult swimming crabs and lesser black-backed gulls (France)	adult swimming crabs and lesser black-backed gulls (Belgium)
0	0.77**	0.50*	0.42	0.52**
1	0.71**	0.45*	0.47	0.54**
2	0.53*	0.22	0.50	0.57**
3	0.42	0.09	0.60*	0.63***
4	0.25	−0.05	0.58*	0.64***

**Figure 2.**
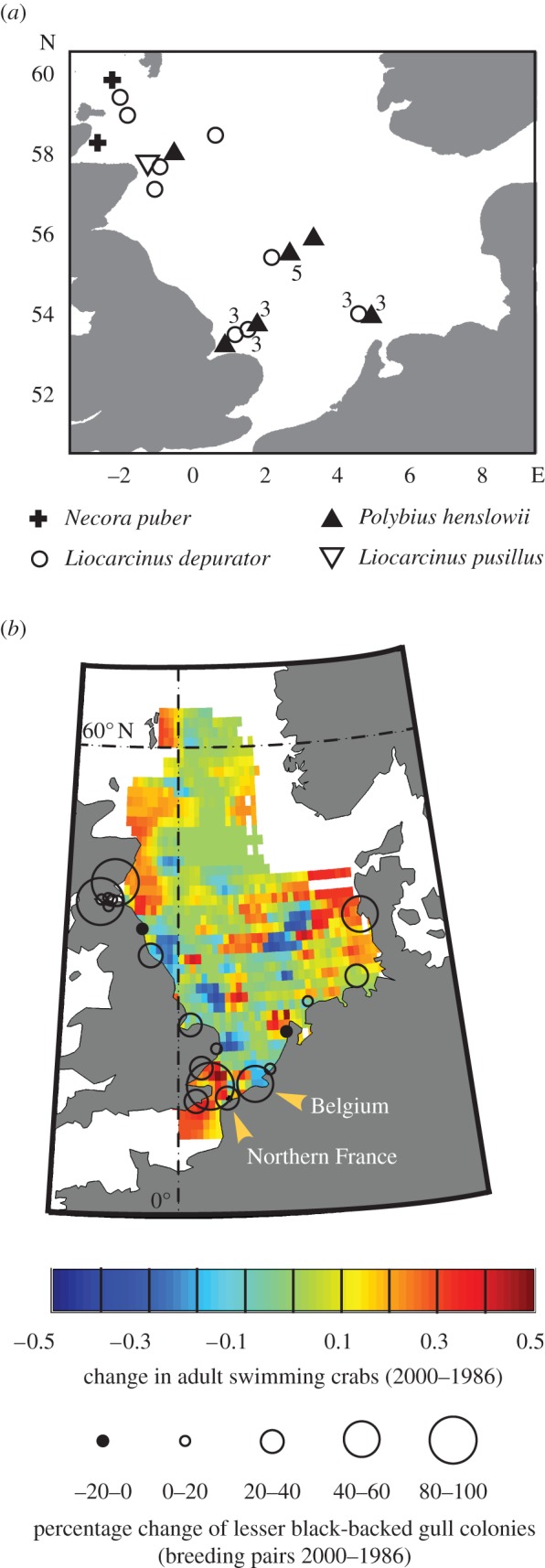
Location of larval swimming crabs in CPR samples and the estimated change between 1986 and 2000 in adult swimming crabs and lesser black-backed gulls. (*a*) Locations of *N. puber*, *P. henslowii*, *L. depurator* and *L. pusillus*. Numbers by symbols indicate the number of larvae of each species when more than one individual was identified. (*b*) Change in the number of swimming crabs and the percentage change of lesser black-backed gull colonies (breeding pairs) (circles). A change of 100% may reflect either the doubling in size of a gull colony, or the appearance of a new colony.

We confirmed the identity of 34 swimming crab larvae in CPR samples, finding three *Necora puber*, 17 *Liocarcinus depurator* and 14 *P. henslowii*. Figure 2*a* shows that the distribution of *P. henlsowii* determined by its larval distribution extends from the southern to the northern North Sea. When we compared the difference in the abundance of adult swimming crabs between 2000 and 1986 with the change in size of 21 lesser black-backed gull colonies, we found an increase in many seagull colonies close to where adult swimming crabs have also increased in abundance (figure 2*b*). A paired *t*-test on the change in adult swimming crabs sampled at the same locations in 1986 and 2000 indicated that the overall increase in adult crabs was statistically significant at the scale of the North Sea (*t* = 2.03 *p* = 0.04, *n* = 157). The Spearman correlation coefficient indicated a significant positive correlation between the changes in adult swimming crabs and the annual population growth rates of lesser black-backed gull colonies (*ρ* = 0.37, *p* < 0.05, *n* = 21).

## 4. Discussion

We provide evidence of a positive correlation between North Sea SST, the abundance of swimming crabs and changes in the abundance of lesser black-backed gulls at 21 major North Sea breeding colonies. In particular, the cross-correlation analyses (table 1) revealed a propagation of a climate signal from SST through decapod larvae, adult crabs and lesser black-backed gulls with lags that match the biology of each trophic group. Many biological changes have been observed among different trophic levels of the North Sea from phytoplankton to fish, as the North Sea has warmed [1]. Here, we suggest that climate-induced changes in the marine fauna extend to the avian fauna, and so also to the terrestrial food web around seabird colonies.

Seabird breeding success is controlled partially by the abundance, composition and nutritional quality of the prey the parents feed to their chicks on the nest [18]. Pelagic swimming crabs are an important component of the diet of seabirds such as the related yellow-legged gulls [19] and lesser black-backed gulls [5,6,20], and they may be especially important during the breeding season [5,15,21] when they have been suggested to provide a source of calcium for both eggshells and the bone development of chicks [5]. The increase in abundance of swimming crabs in the North Sea, including the arrival of *P. henslowi*, may have therefore influenced the breeding success of lesser black-backed gulls. In this respect, the 3–4 year lag we found between the increase in decapods and seagulls (figure 1*b–d* and table 1) is interesting, since this may putatively reflect the time needed for lesser black-backed gulls to reach reproductive maturity [22].

Previously, we have shown that a range expansion of the Balearic shearwater *Puffinus mauretanicus* can be explained by a climate-induced trophic cascade in their fish prey and the  plankton food web [23]. While fishery bycatch and discards can represent a significant supplement for seabirds such as lesser black-backed gulls [15], discards have declined in recent years [24]. The data we now present indicate that a pronounced change in the North Sea ecosystem after approximately 1997, with respect to SST, decapod larvae (predominantly swimming crabs) and adult swimming crabs, was followed shortly by an increase in the size of lesser black-backed gull colonies (figures 1*a–d* and 2*b*).

The inclusion of lesser black-backed gulls in the climate-driven changes of the North Sea ecosystem therefore links marine ecosystem change to terrestrial ecology. On islands, changes in allochthonous nutrient inputs from seabirds have been shown to structure the whole food web [25–27]. Consequently, we suggest that climate-driven changes in the marine food web may also have ensuing ramifications for the coastal ecology of the North Sea region.
